# Antineutrophil cytoplasmic antibody-associated vasculitis with alveolar hemorrhage and ruptured renal aneurysm: A case report and literature review

**DOI:** 10.1097/MD.0000000000028543

**Published:** 2022-01-07

**Authors:** Jin Tong, Zhi-Yu Zhou, Xi Liu, Dao-Xin Wang, Wang Deng

**Affiliations:** a Department of Respiratory and Critical Care Medicine, Second Affiliated Hospital of Chongqing Medical University, Chongqing, China; b Chongqing Medical Research Center for Respiratory and Critical Care Medicine, Chongqing, China; c Department of Interventional Radiology, Second Affiliated Hospital of Chongqing Medical University, Chongqing, China.

**Keywords:** alveolar hemorrhage, ANCA-associated vasculitis, case report, granulomatosis with polyangiitis, PR3-ANCA, renal aneurysm

## Abstract

**Rationale:**

Antineutrophil cytoplasmic autoantibody (ANCA)-associated vasculitis (AAV) is characterized by necrotizing damage to small-vessel vasculitis and mainly occurs in the kidney or lung. We report a rare case of AAV manifesting as alveolar hemorrhage and a renal aneurysm.

**Patient concerns:**

A 50-year-old Chinese man presented with repeated coughing, expectoration, fever, hypoxemia, and respiratory failure. The patient suffered from rupture of the renal aneurysm during immunosuppressive therapy.

**Diagnosis:**

Considering the clinical picture (fever, progressive hypoxemia, renal insufficiency, hemorrhagic bronchoalveolar lavage fluid, and left retroperitoneal hematoma) along with cANCA-PR3 positivity, and lung biopsy findings, the patient was finally diagnosed with granulomatosis with polyangiitis complicated by alveolar hemorrhage and renal aneurysm.

**Interventions:**

The patient was initially treated with immunosuppressive therapy combined with plasma exchange and subsequently with renal arterial embolization due to rupture of the renal aneurysm.

**Outcomes:**

The general condition and inflammatory reaction improved with immunosuppressive therapy combined with plasma exchange. Unfortunately, the patient did not respond to treatment and eventually died of respiratory failure and acute kidney injury after the rupture of the renal aneurysm.

**Lessons:**

We encountered unprecedented difficulties and challenges with renal aneurysm rupture. The possibility of aneurysmal rupture should be carefully considered and frequently checked for immunosuppressive therapy for AAV.

## 1 Introduction

Antineutrophil cytoplasmic autoantibody (ANCA)-associated vasculitis (AAV) is a group of autoimmune disorders characterized by necrotizing damage of small-vessel vasculitis and involves the kidney or lung.^[1]^ AAV is usually associated with cytoplasmic proteins (proteinase3 and myeloperoxidase) expressed in the cytoplasm of neutrophils without an immune complex, according to the Chapel Hill Consensus Conference in 2012.^[2]^ Patients with AAV develop inflammatory necrosis of many organs such as acute respiratory failure (ARF), an uncommon cause that results in mortality.^[3]^

Unlike AAV, polyarteritis nodosa (PAN) typically affects medium-vessel arteries without glomerulonephritis or vasculitis in arterioles, capillaries, or venules. Renal aneurysms are frequently encountered in PAN.^[4]^ ANCA is not typically associated with PAN.^[5]^ Herein, we report a case of PR3-AAV with alveolar hemorrhage and renal aneurysm, typically found in PAN, suggesting that AAV may have some relationship with these complications.

## 2 Case presentation

A 50-year-old Chinese man was admitted for repeated cough, expectoration, fever for 20 days, and aggravation with shortness of breath for half a month. Twenty days ago, the patient presented with repeated cough and expectoration with fever ranging from 38°C to 39°C. In the past half month, the patient's symptoms did not resolve. He presented with shortness of breath, and he was admitted to a local hospital on May 13, 2020. He was intravenously injected with piperacillin (4 g/d, twice a day for 14 days) for anti-infection. Symptoms of fever and shortness of breath progressively worsened. He was transferred to our hospital because of ARF on May 29, 2020. The patient had no relevant medical history.

On physical examination, the patient was 175 cm tall and weighed 76.8 kg. He had a high fever with a body temperature of 38.6°C. He presented with shortness of breath with a respiratory rate of 32 beats/min, pulse rate of 116 beats/min, and blood pressure of 138/88 mm Hg. Some moist rales were heard in the basic areas of both lungs. Results of cardiovascular, abdominal, and musculoskeletal examinations were normal.

**Figure 1:**
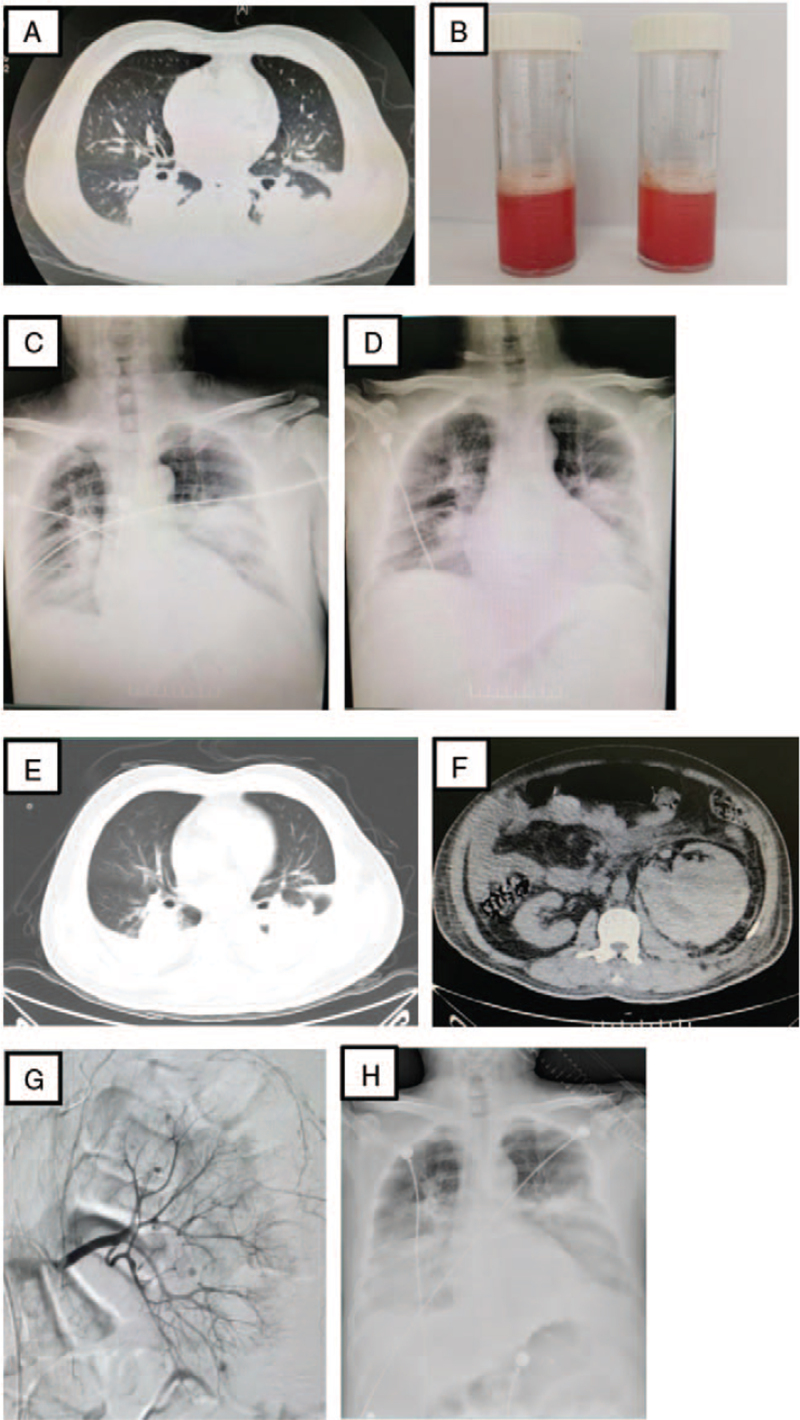
Imaging examinations during hospitalization. (A) On admission, chest X-ray showed bilateral infiltrates in the lower lobe of the lung. (B) Hemorrhagic bronchoalveolar lavage fluid by fiberoptic bronchoscopy. (C) On day 3 of admission, increased lung infiltrates were seen on chest X-ray. (D) On day 6 of admission, bilateral infiltrates in the lung were improved. (E) On day 10 of admission, increased bilateral infiltrates in the lower lobe of the lung were seen on the chest CT scan. (F) Abdominal CT scan showed left kidney and left retroperitoneal hematoma. (G) Selective arterial angiography showed multiple aneurysms in renal arteries. CT = computed tomography.

On admission, arterial blood gas analysis (FiO_2_ 40%) revealed the following: PH 7.49, PCO_2_ 30 mm Hg, PO_2_ 79 mm Hg, PaO_2_/FiO_2_ 197 mm Hg, HCO^3-^ 22.9 mmol/L, and oxygen saturation, 89%. Routine blood tests showed a white blood cell count of 34.82 × 10^9^/L (neutrophils, 95.6%; lymphocytes, 3.1%; eosinophils, 0.6%), platelet count 458 × 10^9^/L, hemoglobin 107 g/L, C-reactive protein 12.36 mg/L. The erythrocyte sedimentation rate was 80 mm/h. Serum creatinine and blood urea nitrogen (BUN) levels were 103 μmol/L and 9.3 mmol/L, respectively, and D-dimer level was 2907 ng/mL. The pro-B-type natriuretic peptide (BNP) level was 6687 pg/mL. His serum procalcitonin (PCT) level was 0.297 ng/mL. Serum K, Na, and Cl were 4.18 mmol/L, 134.8 mmol/L, and 96.9 mmol/L, respectively. The glomerular filtration rate (GFR) and creatinine clearance rate (CCr) were 72.2 mL/min and 70.1 mL/min, respectively. Urinalysis showed 91 leukocytes per μL (0–25) and 337 erythrocytes per μL (0–25). Urinary sediments included erythrocytes 0.3/HP (0–3), leukocytes 2/HP (0–5), and no casts. His IgG and IgE levels were normal. Blood culture and serological tests were negative for human immunodeficiency virus, hepatitis B virus, hepatitis C virus, Epstein-Barr virus, cytomegalovirus, and herpes simplex virus. Chest computed tomography (CT) showed bilateral infiltrates in the lower lobe of the lung (Fig. 1A). The patient was diagnosed with severe pneumonia and ARF. Non-invasive ventilation was performed because of hypoxemia. The patient received intravenous antibiotic treatment (imipenem, 500 mg, 3 times a day).

On day 3 after admission, his shortness of breath and persistent high fever gradually aggravated. Oliguria is also observed. Diffuse airway bleeding was found with hemorrhagic bronchoalveolar lavage fluid on fiberoptic bronchoscopy (Fig. 1B). Autoimmune antibody spectrum were as follows: cytoplasmic staining pattern (cANCA) 1:45 (<1:20), anti- proteinase-3 antibody (PR3-ANCA) 300 RU/mL (0–20), perinuclear staining pattern (pANCA) <1:20 (<1:20), anti-myeloperoxidase antibody (MPO-ANCA) 1.5 RU/mL (0–20), anti-ds-DNA antibody 31.5 IU/mL (0–100), anti-ss-DNA antibody 6.7 IU/mL (0–20), rheumatoid factor 12.06 IU/mL (0–14). Routine blood tests showed a white blood cell count of 32.15 × 10^9^/L (neutrophils 90.2%, lymphocytes 4.3%, eosinophils 0.4%), hemoglobin 99 g/L. The serum creatinine and BUN levels were 301 μmol/L and 19.08 mmol/L, respectively. The GFR and CCr were 19.8 mL/min and 20.3 mL/min, respectively. The serum K, Na, and Cl were 4.13 mmol/L, 132.1 mmol/L, and 96 mmol/L, respectively. The D-dimer level was 3592 ng/mL. pro-BNP was 7745 pg/mL. PCT level was 1.44 ng/mL. Chest radiography revealed an increase in lung infiltrates (Fig. 1C). PR3-AAV with renal insufficiency is also considered. The patient received prednisolone (0.6 mg/kg/day) and cyclophosphamide (200 mg, twice a week) intravenously. Hemodialysis and plasma exchange (PE) were immediately initiated because of deterioration of renal function and alveolar hemorrhage.

**Figure 2:**
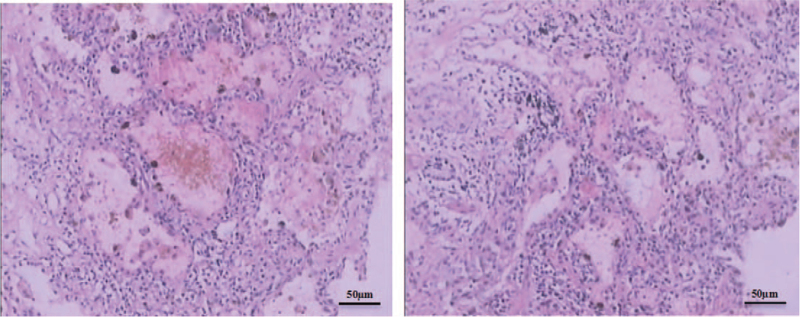
A lung biopsy showed pulmonary capillaritis with extravasation of erythrocytes, fibrosis proliferation and neutrophil infiltration. Length of the scale bar is 50 μm.

His general condition and inflammatory reaction improved with satisfaction from the current treatment. On day 6 after admission, chest radiography revealed improved lung infiltrates (Fig. 1D). A lung biopsy was performed in the presence of pulmonary capillaritis with extravasation of erythrocytes, fibrotic proliferation, and neutrophil infiltration (Fig. 2). On day 10 after admission, the patient had a sharp pain of sudden onset in his left abdominal side. His hemoglobin level decreased from 99 g/L to 55 g/L developed in the subsequent hours, but there was no sign of hemorrhage. Chest CT revealed increased bilateral infiltrates in the lower lobe of the lung (Fig. 1E). Abdominal CT revealed a left kidney and a left retroperitoneal hematoma (Fig. 1F). Selective arterial angiography revealed multiple aneurysms of the renal arteries (Fig. 1G). A decrease in hemoglobin and a large hematoma in the left kidney and left retroperitoneum were considered the possibility of a ruptured renal aneurysm based on arterial angiography. Selective renal arterial embolization was performed. The patient was diagnosed with granulomatosis and polyangiitis (GPA) complicated by alveolar hemorrhage and renal aneurysm.

**Figure 3:**
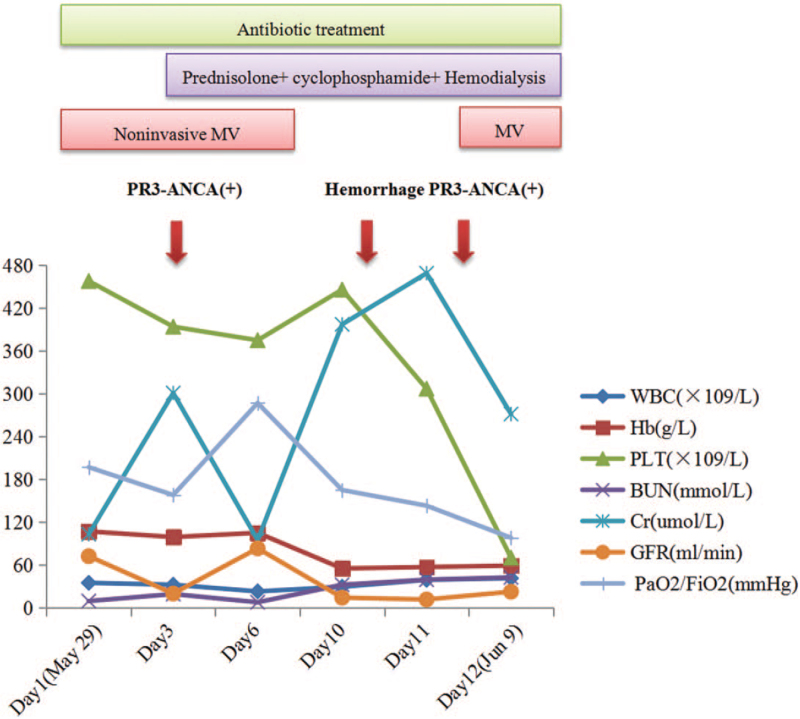
Treatment of the patient during hospitalization.

On day 11 after admission, his shortness of breath and renal function gradually deteriorated, and serum creatinine and BUN levels were 469 μmol/L and 39.26 mmol/L, respectively. GFR and CCr were11.5 mL/min and 12.2 mL/min, respectively. Serum K, Na, and Cl were 5.92 mmol/L, 138.3 mmol/L, and 101.1 mmol/L, respectively. The pro-BNP was 33568 pg/mL. The white blood cell count 39.03 × 10^9^/L (neutrophils 92.8%, lymphocytes 4%, eosinophils 0%), platelet count 307 × 10^9^/L, hemoglobin 57 g/L, and PCT level 7.13 ng/mL. Arterial blood gas analysis indicated hypoxemia with a PaO_2_/FiO_2_ ratio of 143 mm Hg. cANCA and PR3-ANCA were 1:100 and 264 RU/mL, respectively. Chest radiography revealed an increase in lung infiltrates (Fig. 1H). Intubation with mechanical ventilation was performed immediately. Unfortunately, the patient died of ARF and acute kidney injury (AKI) on June 9, 2020 (Fig. 3).

## 3 Literature review and discussion

AAV is characterized by small-vessel vasculitis, particularly involving the glomerular and pulmonary capillaries, that leads to rapidly progressive glomerulonephritis and pulmonary hemorrhage (more than 50%).^[6]^ In this case, white lung with consolidations, alveolar hemorrhage, mild normocytic anemia, and cANCA-PR3 positivity were considered as diagnoses of GPA. In detail, the patient suffered from multiple organ damage, particularly ARF caused by severe lung involvement and rapidly progressive AKI with a rare involvement of a renal aneurysm that is difficult to treat.

Although the lung is the most commonly affected organ in GPA, alveolar hemorrhage and ARF are rare clinical manifestations that carry an extremely high mortality rate, with an incidence of only 5% to 10% in these patients.^[7–11]^ High-risk factors, including infections, environment, or genetic predisposition, may trigger an inflammatory response that involves the release of ANCA.^[12]^ ANCA-activated neutrophils secrete proinflammatory cytokines and release reactive oxygen species to amplify vasculitis and disrupt the vascular wall, migrate into lung compartments through the alveolar-capillary membrane, and finally reach alveolar spaces mediated by CXC chemokines.^[13,14]^ In addition, severe damage to the alveolar-capillary barrier can cause diffuse intra-alveolar hemorrhage from small vessels, which is an important predictor of ARF in patients with AAV.^[15,16]^ In this case, the patient's respiratory failure progressively developed due to pulmonary hemorrhage despite the use of non-invasive mechanical ventilation. Immunosuppressive therapy combined with PE was performed according to the European vasculitis study group recommendations.^[17]^ The patient's condition dramatically improved due to supportive therapies for complications affecting the vital organs.

Arterial aneurysm formation and rupture are rare complications of GPAs. To the best of our knowledge, only cases with sufficient clinical data for analysis have been identified through a literature review. Finally, 6 case reports with renal aneurysm involvement were included in the present study (Table 1). All patients were men, and the age of onset ranged from 24 to 50 years. Three patients showed positive ANCA results. Moreover, 3 patients had ruptured renal aneurysms, of which 1 died. In addition, most of these patients were treated with immunosuppressive agents, such as steroids and cyclophosphamide. The prognosis of surviving patients was good.

**Table 1:** Characteristics of patients with renal aneurysm in granulomatosis with polyangiitis.

Case/year	Gender/age (yr)	Country	Manifestation	Antibodies	Symptoms	Duration of aneurysmal symptoms	Complication with alveolar hemorrhage	Treatment	Rupture	Outcome
Baker/1978	Male/24	American	Renal aneurysms	NS	Abdominal pain	6 wks	No	PSL 30 mg + CY 150 mg/day	Yes	Good
Moutsopoulos/1983	Male/30	Greece	Renal aneurysms	NS	NS	1 mo	No	PSL 1 mg/kg/day + CY 2 mg/kg/day	No	Good
Senf/2003	Male/35	Germany	Hepatic, renal, splanchnic aneurysms	C-ANCA positive	Abdominal pain	6 wks	No	Steroid + PSL + IVCY 750 mg	Yes	Good
Arlet/2008	Male/29	France	Hepatic + renal aneurysm	PR3 ANCA 15 IU/mL	Abdominal pain	NS	No	Coil embolization + steroid + PSL60 mg + MMF 2.5 g	No	Good
Unlü/2011	Male/43	Netherlands	Renal aneurysms	NS	Abdominal pain and generalized malaise	1 wk	No	PSL + surgery	No	Good
Present case	Male/50	China	Renal aneurysms	c-ANCA + PR3 positive	Abdominal pain	3 h	Yes	Embolization + PSL + CY200 mg + hemodialysis	Yes	Death

It is difficult to prove the relationship between GPA and renal aneurysm because of the lack of pathological evidence in our patient. This finding suggests that the incidence of both PR3-AAV and renal aneurysm are very remote by chance through their pathologies. It is more reasonable to consider that the pathology of PR3-AAV is related to the development of renal aneurysms. Aneurysms of medium-sized arteries are frequently encountered in PAN, but their occurrence in smaller arteries remains unclear. Damage to the internal and external elastic lamina may contribute to development of aneurysmal dilation.^[18]^ Additionally, the vessel wall is disrupted by inflammation, thrombosis, and arterial narrowing, resulting in aneurysm formation. Immunosuppressive therapies could lead to thinning of the adventitia, which requires consideration of the risk of aneurysmal rupture.^[19]^

In the present case, the patient developed both alveolar hemorrhage and a renal aneurysm, a rare manifestation of GPA. PE is recommended in the presence of alveolar hemorrhage and severe renal disease, which can effectively remove ANCAs and yield a good prognosis.^[20]^ Corticosteroids and cyclophosphamide are reportedly effective for the treatment of arterial aneurysms in GPA. Unfortunately, the patient's condition progressively deteriorated, with ARF and AKI. Aneurysmal rupture may result in life-threatening bleeding and poor outcomes. ANCA might play a role in ANCA-associated large-vessel disease in rare cases.^[21]^ Large-vessel involvement should be considered an important factor in the development of AAV. Therefore, CT scans should be considered when screening for the presence of an arterial aneurysm to treat the patient as safely as possible. There are valuable experiences and lessons learned from cases where rare manifestations of rare diseases are encountered in the process of clinical diagnosis.

## 4 Conclusion

In conclusion, GPA can be life-threatening, especially in cases involving large-vessel vasculitis, which should not be ignored by clinicians. Only scarce reports of GPA with alveolar hemorrhage and ruptured renal aneurysm have been reported. The possibility of aneurysmal rupture should be carefully considered and the condition of the aneurysm should be checked frequently when administering immunosuppressive therapies for GPA with aneurysm.

## Author contributions

**Conceptualization:** Jin Tong, Wang Deng.

**Data curation:** Jin Tong, Wang Deng.

**Formal analysis:** Zhi-Yu Zhou, Xi Liu.

**Funding acquisition:** Wang Deng.

**Resources:** Jin Tong, Wang Deng.

**Supervision:** Wang Deng.

**Writing – original draft:** Jin Tong.

**Writing – review & editing:** Dao-Xin Wang, Wang Deng.
